# Screening of Key Pathways and Key Genes for the Differential Regulation of Subcutaneous and Intramuscular Fat Deposition by FTO in Chickens

**DOI:** 10.3390/cells15100903

**Published:** 2026-05-14

**Authors:** Hua-Yun Huang, Yi Kong, Chun-Miao Li, Yu-Le Sui, Qian-Bao Wang, Zhen-Hua Zhao, Ling-Lin Kong, Zhao-Lin Wu, Wei Han

**Affiliations:** Institute of Poultry Science, Yangzhou 225125, China; huanghuayun520@163.com (H.-Y.H.);

**Keywords:** fat mass and obesity-associated gene, chicken, fat deposition

## Abstract

The fat mass and obesity-associated gene (*FTO*) has been shown to play a critical role in fat deposition in both humans and livestock. However, its involvement in subcutaneous and intramuscular fat deposition in chickens remains underexplored. In this study, we investigated the regulatory effects and pathways of *FTO* on subcutaneous and intramuscular fat deposition in chickens through functional gene verification and bioinformatics analysis. Our results demonstrated that, compared to the control group, exogenous transfection of an *FTO* lentiviral overexpression vector significantly inhibited cell proliferation and increased lipid accumulation in both subcutaneous and intramuscular adipocytes (*p* < 0.05). Furthermore, transfection of *FTO* siRNA markedly increased cell proliferation and reduced lipid accumulation in both subcutaneous and intramuscular adipocytes. A total of 413 and 164 differentially expressed genes were regulated by *FTO* in subcutaneous and intramuscular adipocytes, respectively. Pathway analysis revealed that the regulation of the actin cytoskeleton was a key process involved in *FTO*-mediated fat deposition in both subcutaneous and intramuscular adipocytes. Additionally, *NRAS* and *ITGAV* (subcutaneous fat), as well as *FGF9*, *PIK3R2*, *FGF16*, and *RHOA* (intramuscular fat), were identified as key genes enriched in this pathway. In conclusion, *FTO* differentially regulates fat deposition in chicken subcutaneous and intramuscular adipocytes by targeting distinct functional genes within the actin cytoskeleton pathway.

## 1. Introduction

In the poultry industry, fat traits have long been a major research focus due to their strong association with key factors such as feed conversion ratio, meat type, and meat quality, particularly flavor. In chickens, body fat primarily comprises subcutaneous fat, abdominal fat, and intramuscular fat. Intramuscular fat, which is located within muscle fibers and between muscle bundles, plays a vital role in muscle composition. As an energy reserve, it has a significant impact on meat quality characteristics, including tenderness, flavor, and juiciness. Research has demonstrated that adequate subcutaneous fat deposition helps retain moisture in meat, thereby preserving its softness and tenderness during cooking. Furthermore, increased fat content contributes to enhanced juiciness and a richer flavor profile [1]. Therefore, identifying genes that are strongly associated with subcutaneous and intramuscular fat deposition will provide a theoretical foundation for molecular breeding strategies targeting these complex traits, as well as offering valuable insights into the molecular mechanisms governing the deposition of these two types of fat.

The fat mass and obesity-associated gene (*FTO*) was the first gene identified as being strongly associated with human obesity through genome-wide association studies (GWAS). Recent studies across various species have demonstrated that *FTO* plays a pivotal role in fat deposition by regulating RNA methylation, adipocyte differentiation, and energy metabolism pathways. Additionally, *FTO* is critical in fatty acid transport [2]. Studies have shown that human preadipocytes carrying the *FTO* allele (rs1421085 CC) exhibit significantly reduced UCP1 expression and mitochondrial respiration during differentiation into beige adipocytes [3,4]. In human studies, *FTO* overexpression promotes adipogenesis and lipid storage, inhibits lipolysis through the SREBP1c pathway, and accelerates excessive lipid accumulation in the liver [5]. In mice, *FTO* overexpression increases appetite and contributes to obesity [6,7], while *FTO* knockout suppresses fat deposition in the liver [8]. In pigs, *FTO* knockdown significantly increases the m6A methylation level of total RNA, reducing lipid accumulation in both porcine adipocytes and 3T3-L1 preadipocytes [9]. In goats, *FTO* affects mitochondrial content and lipid metabolism by regulating N6-methyladenosine (m6A) modification [10]. In cattle, *FTO* mutations are significantly associated with lean meat percentage [11]. Collectively, these findings indicate that *FTO* is strongly associated with obesity in humans and fat deposition in mammals.

In poultry, our previous study demonstrated that *FTO* promotes chicken myoblast differentiation through focal adhesion-mediated signaling pathways [12]. Myocytes and adipocytes maintain a dynamic balance in tissue development through competition and mutual regulation. Building on this, we hypothesized that *FTO* may also play a role in regulating intramuscular fat deposition in chickens. In this study, we constructed an *FTO* lentiviral overexpression vector and integrated functional gene verification with bioinformatics analysis to identify the key genes and signaling pathways through which *FTO* regulates lipid deposition in both subcutaneous and intramuscular adipocytes. Our findings aim to provide a theoretical foundation for further elucidating the molecular mechanisms underlying fat deposition in different tissues of chickens.

## 2. Materials and Methods

All animal experimental procedures were performed in accordance with the guidelines for the care and use of experimental animals established by the Ministry of Agriculture and Rural Affairs of the People’s Republic of China. The collection of cell and tissue samples was approved by the Ethics Committee of the Scientific Research Department at the Jiangsu Institute of Poultry Science (Approval No. JQ20230920).

### 2.1. Isolation and Culture of Primary Chicken Subcutaneous and Intramuscular Adipocytes

Subcutaneous adipose tissue was collected from newly hatched yellow-feathered broilers, carefully dissected to remove fascia, minced, and digested with 0.1% type I collagenase in a 37 °C incubator for 1 h. Digestion was terminated by the addition of 10% complete medium. The resulting cell pellet was then harvested, resuspended, and cultured at 37 °C. Adipocyte differentiation was induced by the addition of 0.1% oleic acid.

Intramuscular adipocytes were isolated from the pectoralis muscle of newly hatched yellow-feathered broilers. The tissues were digested with 0.1% type I collagenase at 37 °C for 2 h, with gentle mixing every 20 min. After centrifugation, the supernatant was collected and cultured at 37 °C. Adipocyte differentiation was induced by the addition of 0.1% oleic acid.

### 2.2. Construction of FTO Lentiviral Expression Vector

The *FTO* lentiviral expression vector was constructed by Genepharma Co., Ltd. (Suzhou, China). Based on the CDS sequence of chicken *FTO* deposited in GenBank (NM_001077232.2), the target gene was synthesized through whole-gene synthesis. The full-length *FTO* CDS was digested with NotI and NsiI, purified, and ligated into the vector. The recombinant plasmid was then transformed into TOP10 competent cells. Positive clones were confirmed by sequencing, and clones with 100% sequence identity were selected as the correct *FTO* expression vector.

The recombinant *FTO* plasmid was ultra-purified and used for lentivirus production. The packaging plasmids (pGag/Pol, pRev, pVSV-G) and the *FTO* expression plasmid were co-transfected into 293T cells. Lentiviral particles were harvested, concentrated, and the viral titer was determined. The titer of the *FTO* lentiviral expression vector was 2 × 10^8^ TU/mL. 

### 2.3. Lentiviral Transfection and Cell Treatment

Subcutaneous and intramuscular adipocytes were transfected with either the *FTO* lentiviral expression vector or a negative control vector at 40–50% confluence in 6-well plates. Polybrene (5 μg·mL^−1^) was added to enhance transfection efficiency. At 72 h post-transfection, green fluorescence was observed under an inverted fluorescence microscope, and *FTO* mRNA expression was analyzed by quantitative real-time PCR (qPCR). Each group included three biological replicates.

Cell proliferation was assessed at 72 and 96 h post-transfection using a CCK-8 kit (Dojindo Laboratories, Fukuoka, Japan) and Edu kit (Ruibo Biotech Co., Ltd., Guangzhou, China), with six replicates per group. For lipid deposition analysis, cells were treated with 0.1% oleic acid for 6 days, beginning 1 day after transfection. Lipid droplet accumulation was evaluated by Oil Red O staining, with three replicates per group.

### 2.4. FTO siRNA Transfection and Cell Treatment

The *FTO* siRNA sequence was synthesized by GenePharma (Shanghai, China). Based on the mRNA sequence of chicken *FTO* (NM_001185147.1), three pairs of siRNA primers were designed using Oligo Designer version 3.0 (Table 1) and the *FTO*-858 primer was validated in vitro.

Cell proliferation was assessed at 24, 48 and 72 h post-transfection using a CCK-8 kit (Dojindo Laboratories, Fukuoka, Japan), with six replicates per group. For lipid deposition analysis, cells were treated with 0.1% oleic acid for 4 days, beginning 1 day after transfection. Lipid droplet accumulation was evaluated by Oil Red O staining, with three replicates per group.

### 2.5. Total RNA Isolation, Primer Synthesis, Reverse Transcription and qPCR

Total RNA was extracted from cells using the RNAprep Pure Kit (DP419, Tiangen Biotech Co., Ltd., Shanghai, China) according to the manufacturer’s instructions.

Gene-specific primers were designed using Oligo software and synthesized by Sangon Biotech Co., Ltd. (Shanghai, China). cDNA synthesis was performed using a reverse transcription kit (RR036Q, Takara, Dalian, China).

Quantitative real-time PCR was conducted using the QuantiNova SYBR Green PCR Kit (4993626, QIAGEN, Hilden, Germany) following the manufacturer’s protocol. Primer sequences are provided in Table 1.

### 2.6. Transcriptome Sequencing Analysis

Transcriptome sequencing was performed on cells transfected with the *FTO* lentiviral expression vector and the negative control vector. All sequencing procedures were carried out by Genedenovo Biotechnology Co., Ltd. (Guangzhou, China) using an Illumina platform. Raw reads were filtered to remove low-quality sequences using fastp (v 0.18.0). Clean reads were then mapped to the reference genome using HISAT2 (v 2.1.0). Gene expression levels were quantified with StringTie (v 1.3.4) and expressed as transcripts per million (TPM). Differentially expressed genes (DEGs) were identified using the edgeR package (v 3.12.1). Genes with an FDR < 0.05 or *p* < 0.05 and fold change (FC) > 1.5 or FC < 0.67 were considered significantly differentially expressed.

GO and KEGG enrichment analyses were conducted using Kobas 3.0. Terms and pathways with *p* < 0.05 were considered significantly enriched. Protein–protein interaction (PPI) analysis was performed using the STRING database (http://string-db.org). The PPI network was then constructed and visualized using Cytoscape 3.8.0.

### 2.7. Statistical Analysis

All data are presented as the mean ± standard deviation (SD). Statistical analyses were conducted using IBM SPSS Statistics version 23.0 (IBM Corp., Armonk, NY, USA), with one-way analysis of variance (ANOVA). Differences were considered statistically significant at *p* < 0.05 and non-significant at *p* > 0.05.

## 3. Results

### 3.1. The Role of FTO in Proliferation and Lipid Droplet Deposition in Chicken Subcutaneous Adipocytes

#### 3.1.1. Effects of FTO Lentiviral Expression Vector Transfection on Subcutaneous Adipocytes Proliferation and Lipid Droplet Accumulation

**Figure 1:**
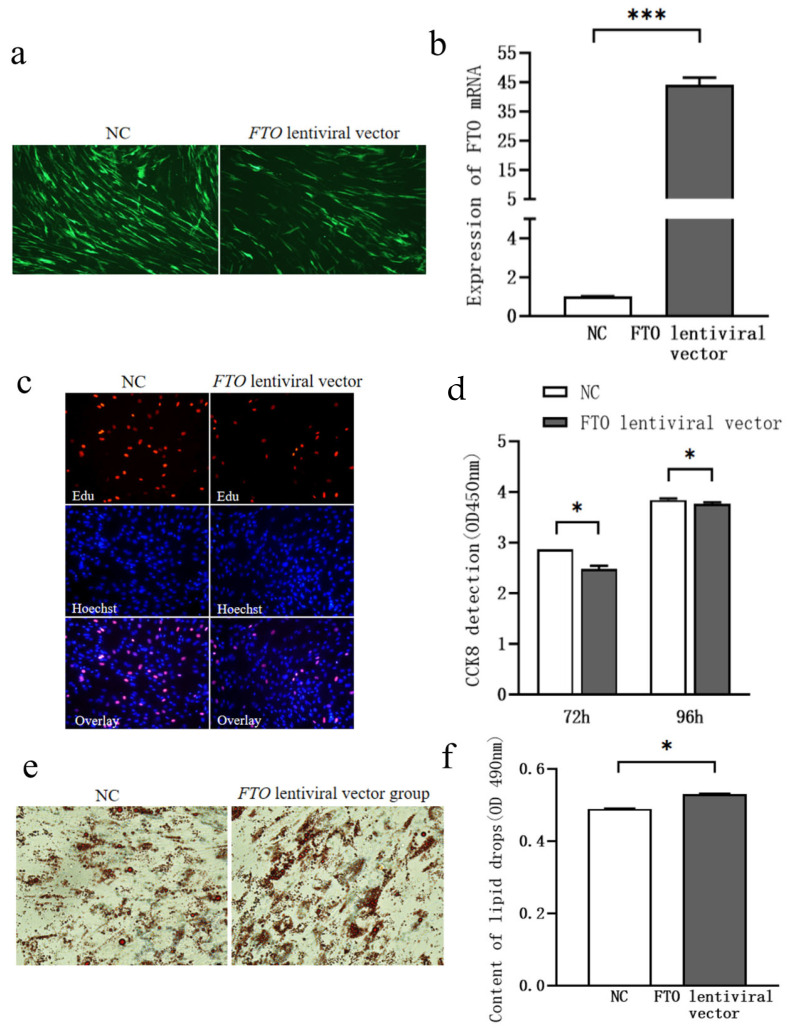
Effect of *FTO* Overexpression on Lipid Deposition in Chicken Subcutaneous Adipocytes. (**a**) *FTO* Lentiviral Expression Vector Transfection (100×); (**b**) Changes in *FTO* mRNA Expression Following Exogenous Transfection of the *FTO* Lentiviral Expression Vector; (**c**) EdU Staining (200×); (**d**) Changes in Cell Proliferation Assessed by CCK-8 Assay; (**e**) Oil Red O Staining (400×); (**f**) Changes in Lipid Droplet Accumulation. * means *p* < 0.05, *** means *p* < 0.001.

Subcutaneous adipocytes were transfected with either the *FTO* lentiviral expression vector or a negative control vector in 6-well plates at 40–50% confluence. As shown in Figure 1a, green fluorescence indicated successful transfection. qPCR analysis demonstrated that *FTO* mRNA expression was significantly higher in the *FTO*-overexpressing group compared to the negative control group at 3 days post-transfection (Figure 1b, *p* < 0.01), confirming efficient transfection of the *FTO* lentiviral vector.

To investigate the role of *FTO* in the proliferation of chicken subcutaneous adipocytes, cells were transfected with either the *FTO* lentivirus vector or a negative control vector. Cell proliferation was assessed using CCK-8 and EdU staining assays. Compared to the control group, cell proliferation was significantly reduced at 72 and 96 h following *FTO* overexpression (Figure 1c,d, *p* < 0.05).

Six days after *FTO* lentiviral transfection, Oil Red O staining revealed that lipid droplet accumulation was significantly higher in the *FTO*-overexpressing group compared to the control group (Figure 1e,f, *p* < 0.05).

#### 3.1.2. Effects of FTO siRNA Transfection on Proliferation and Lipid Droplet Accumulation in Chicken Subcutaneous Adipocytes

To further investigate the role of *FTO* in the proliferation and lipid droplet accumulation of chicken subcutaneous adipocytes, *FTO* siRNA was transfected into the cells, and subsequent changes in cell proliferation and lipid deposition were systematically observed.

**Figure 2:**
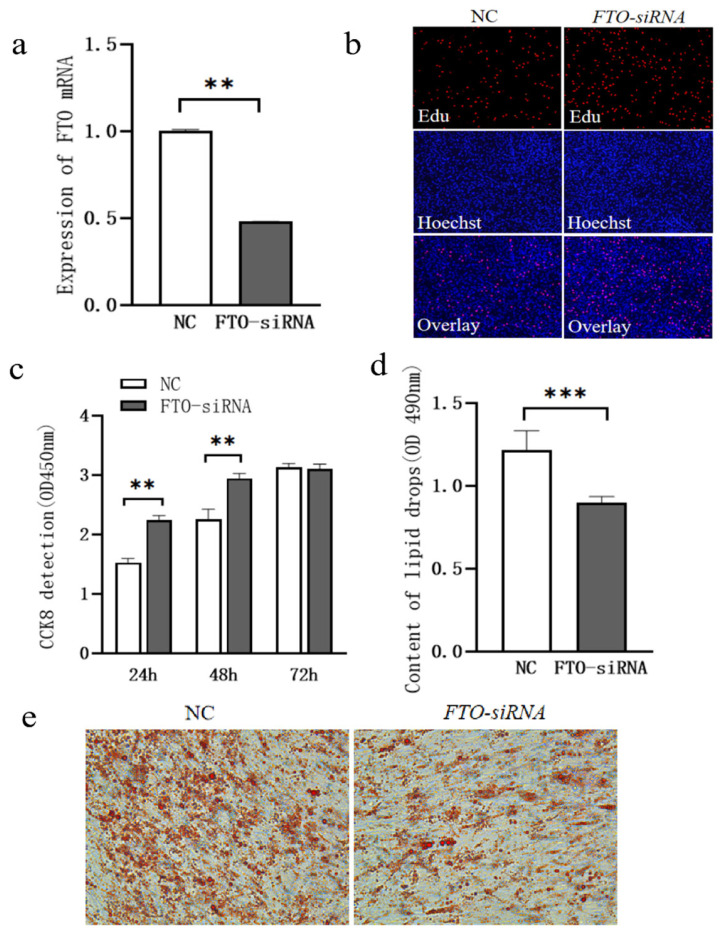
Effect of *FTO* siRNA Transfection on Proliferation and Lipid Droplet Accumulation of Chicken Subcutaneous Adipocytes. (**a**) Changes in *FTO* mRNA Expression Following Exogenous Transfection of *FTO* siRNA; (**b**) EdU Staining (100×); (**c**) Changes in Cell Proliferation Assessed by CCK-8 Assay; (**d**) Changes in Lipid Droplet Accumulation; (**e**) Oil Red O Staining (400×); ** means *p* < 0.01, *** means *p* < 0.001.

After *FTO* siRNA transfection into subcutaneous adipocytes, the mRNA expression of *FTO* was significantly downregulated (Figure 2a, *p* < 0.05). Cell proliferation was assessed using Cell Counting Kit-8 (CCK-8) and 5-ethynyl-2′-deoxyuridine (EdU) staining assays. Compared to the control group, cell proliferation was significantly enhanced at 24 and 48 h post-transfection of *FTO* siRNA (Figure 2b,c, *p* < 0.05). Four days post-transfection of *FTO* siRNA, Oil Red O revealed that the accumulation of lipid droplets in the *FTO* siRNA group was significantly lower than in the control group (Figure 2d, *p* < 0.05).

#### 3.1.3. Screening of Pathways and Genes Underlying FTO-Regulated Lipid Deposition in Chicken Subcutaneous Adipocytes

**Figure 3:**
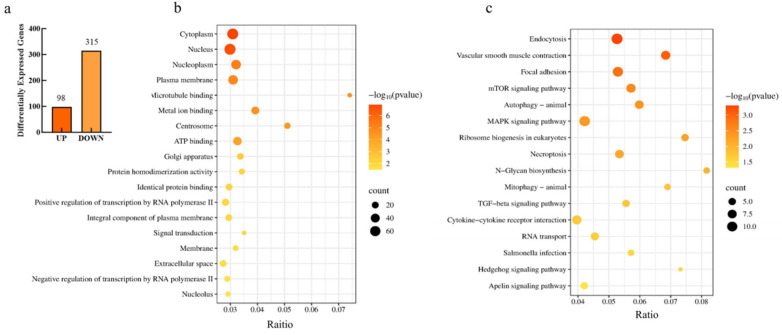
Analysis of Transcriptome Sequencing Data in Subcutaneous Adipocytes. (**a**) Differentially Expressed Genes; (**b**) GO Enrichment; (**c**) KEGG Enrichment.

To further investigate the pathways and genes responsive to *FTO* regulation in subcutaneous adipocytes, transcriptome sequencing was performed on *FTO*-transfected and negative control cells to identify the key genes and pathways regulated by *FTO*. A total of 413 differentially expressed genes (DEGs) were identified, including 98 upregulated genes and 315 downregulated genes (Figure 3a, *p* < 0.05).

To further characterize the functions of the differentially expressed genes (DEGs), Gene Ontology (GO) and Kyoto Encyclopedia of Genes and Genomes (KEGG) enrichment analyses were performed. GO enrichment analysis revealed that 84 GO terms were significantly enriched. The top 20 GO terms primarily included cytosol, cytoplasm, nucleoplasm, integral component of membrane, ATP binding, positive regulation of transcription by RNA polymerase II, and the integral component of plasma membrane (Figure 3b, *p* < 0.05).

KEGG enrichment analysis revealed that 17 signaling pathways were significantly enriched, including regulation of the actin cytoskeleton, focal adhesion, mTOR signaling pathway, autophagy, Apelin signaling pathway, JNK/p38 MAPK signaling pathway, eukaryotic ribosome biogenesis, cytokine–cytokine receptor interaction, N-glycan biosynthesis, and transforming growth factor-β (TGF-β) signaling pathway (Figure 3c, *p* < 0.05). Genes enriched in these pathways are listed in Table 2 (*p* < 0.05).

**Figure 4:**
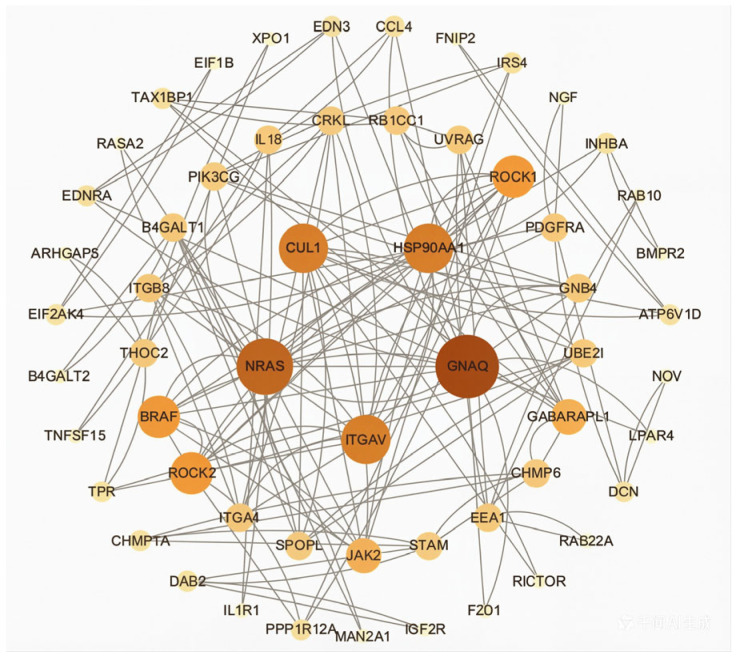
PPI Analysis of Differentially Expressed Genes in Significantly Enriched Pathways of Subcutaneous Adipocytes.

To further identify key genes and pathways responsive to *FTO* regulation, protein–protein interaction (PPI) analysis was performed on the genes within the significantly enriched pathways. As shown in Figure 4, *CUL1*, *NRAS*, *ITGAV*, *GNAQ*, and *HSP90AA1* were identified as key genes involved in *FTO*-mediated lipid deposition in chicken subcutaneous adipocytes.

### 3.2. Regulatory Effect and Pathway of FTO on Proliferation and Lipid Deposition in Chicken Intramuscular Adipocytes

#### 3.2.1. Effects of FTO Lentiviral Expression Vector Transfection on Proliferation and Differentiation of Chicken Intramuscular Adipocytes

**Figure 5:**
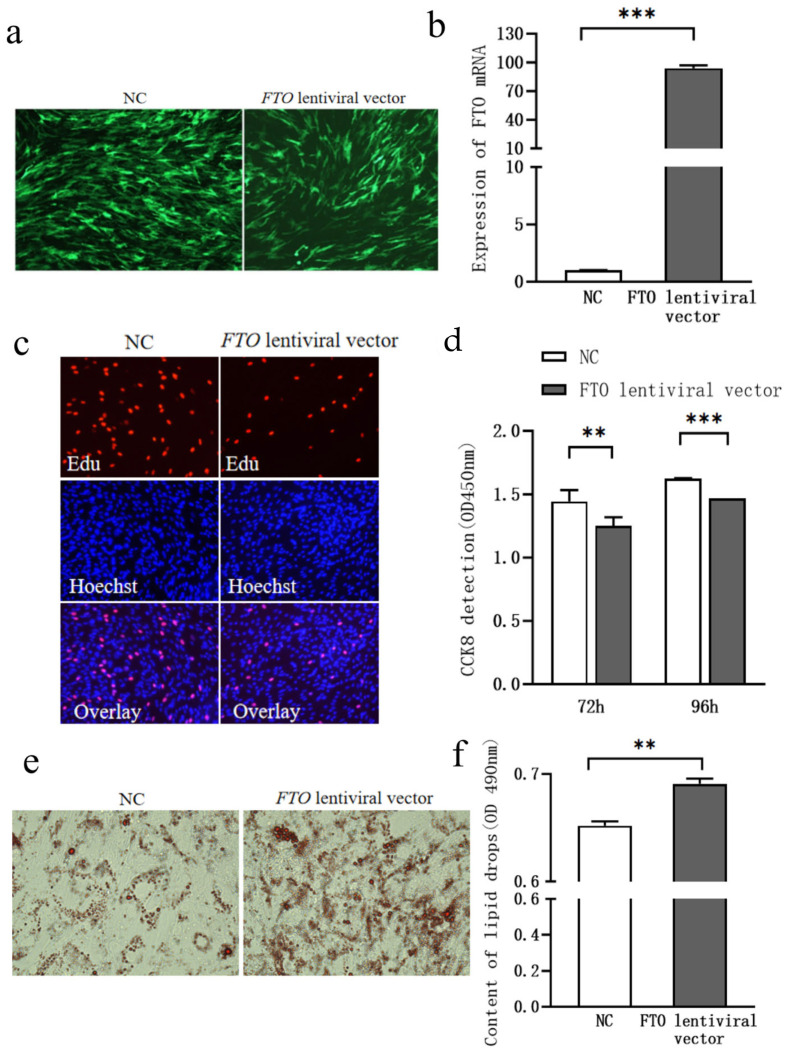
Effect of *FTO* Overexpression on the Proliferation of Chicken Intramuscular Adipocytes. (**a**) *FTO* Lentiviral Expression Vector Transfection (100×); (**b**) Changes in *FTO* mRNA Expression Following Exogenous Transfection of the *FTO* Lentiviral Expression Vector; (**c**) EdU Staining (200×); (**d**) Changes in Cell Proliferation Assessed by CCK-8 Assay; (**e**) Oil Red O Staining (400×); (**f**) Changes in Lipid Droplet Accumulation. ** means *p* < 0.01, *** means *p* < 0.001.

Intramuscular adipocytes were transfected with the *FTO* lentiviral expression vector at 40–50% confluence. As shown in Figure 5a, green fluorescence indicated successful transfection. qPCR analysis revealed that *FTO* mRNA expression was significantly higher in the *FTO*-transfected group compared to the negative control group at 3 days post-transfection (Figure 5b, *p* < 0.01), confirming the successful transfection of the *FTO* lentiviral vector.

Intramuscular adipocytes were transfected with either the *FTO* lentiviral expression vector or the negative control vector. Cell proliferation was assessed using the CCK-8 assay at 72 and 96 h post-transfection. Compared to the control group, cell proliferation was significantly reduced in the *FTO*-overexpressing group at both 72 and 96 h (Figure 5c,d, *p* < 0.05).

Six days after *FTO* lentiviral transfection, Oil Red O staining revealed that lipid droplet accumulation was significantly higher in the *FTO*-transfected group compared to the control group (Figure 5e,f, *p* < 0.05).

#### 3.2.2. Effects of FTO siRNA Transfection on Proliferation and Lipid Droplet Accumulation in Chicken Intramuscular Adipocytes

**Figure 6:**
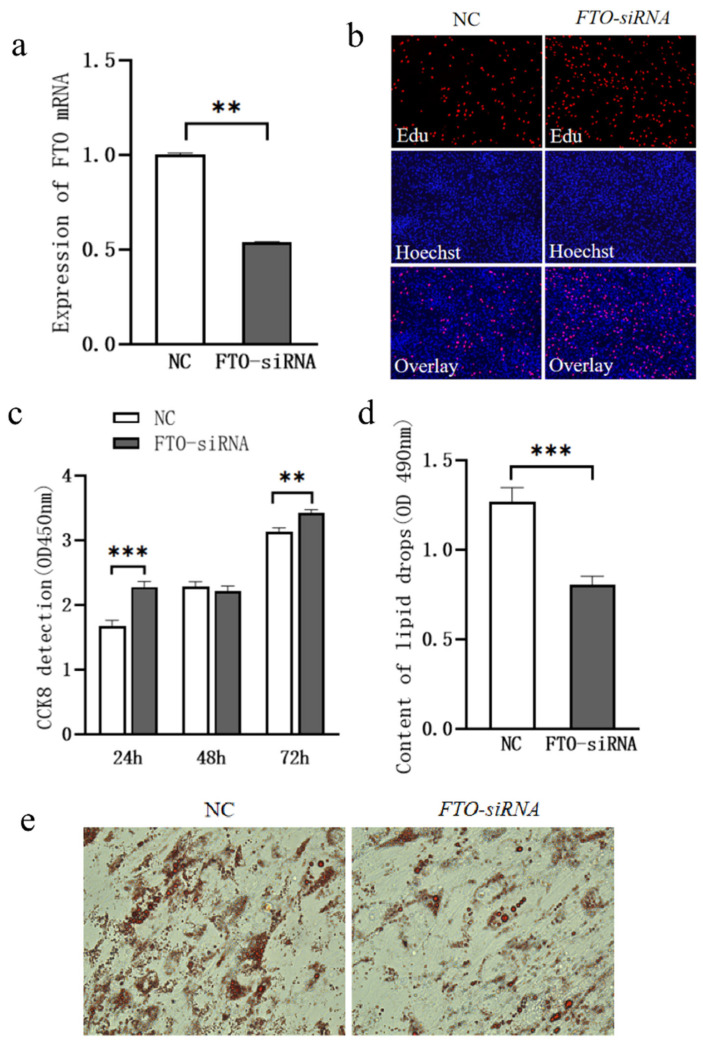
Effect of *FTO* siRNA Transfection on Proliferation and Lipid Droplet Accumulation in Chicken Intramuscular Adipocytes. (**a**) Changes in *FTO* mRNA Expression Following Exogenous Transfection of FTO siRNA; (**b**) EdU Staining (100×); (**c**) Changes in Cell Proliferation Assessed by CCK-8 Assay; (**d**) Changes in Lipid Droplet Accumulation; (**e**) Oil Red O Staining (400×). ** means *p* < 0.01, *** means *p* < 0.001.

To further investigate the role of *FTO* in the proliferation and lipid droplet accumulation of chicken intramuscular adipocytes, *FTO* siRNA was transfected into the cells, and the changes in cell proliferation and lipid deposition were observed. After *FTO* siRNA transfection into intramuscular adipocytes, the mRNA expression of *FTO* was significantly decreased (Figure 6a, *p* < 0.05).

Cell proliferation was assessed using CCK-8 and EdU staining assays. Compared to the control group, cell proliferation was significantly enhanced at 24 and 72 h after *FTO* siRNA transfection (Figure 6b,c, *p* < 0.05); Four days after *FTO* siRNA transfection, Oil Red O staining revealed that lipid droplet accumulation was significantly lower in the *FTO*-siRNA group compared to the control group (Figure 6d, *p* < 0.05).

#### 3.2.3. Screening of Pathways and Genes Underlying FTO-Regulated Lipid Deposition in Intramuscular Adipocytes

**Figure 7:**
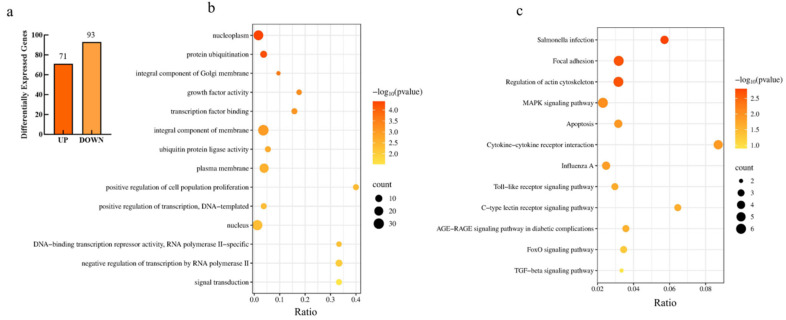
Analysis of Transcriptome Sequencing Data in Intramuscular Adipocytes. (**a**) Differentially Expressed Genes; (**b**) GO Enrichment; (**c**) KEGG Enrichment.

To further investigate the molecular mechanism by which *FTO* regulates lipid droplet accumulation in intramuscular adipocytes, transcriptome sequencing was performed on *FTO*-transfected and negative control cells to identify genes and pathways responsive to *FTO* regulation. A total of 164 differentially expressed genes (DEGs) were identified between the two groups, including 71 upregulated and 93 downregulated genes (Figure 7a, *p* < 0.05).

To further characterize the functions of the differentially expressed genes (DEGs), Gene Ontology (GO) and Kyoto Encyclopedia of Genes and Genomes (KEGG) enrichment analyses were performed. GO enrichment analysis revealed 54 significantly enriched GO terms, including 15 cellular component (CC) terms, 11 molecular function (MF) terms, and 28 biological process (BP) terms. The top five terms in each category are shown in Figure 7b (*p* < 0.05).

KEGG analysis revealed that 13 signaling pathways were significantly enriched, including focal adhesion, regulation of the actin cytoskeleton, MAPK signaling pathway, gap junction, cytokine–cytokine receptor interaction, FoxO signaling pathway, apoptosis, C-type lectin receptor signaling pathway, and TGF-β signaling pathway (Figure 7c, *p* < 0.05). DEGs enriched in these pathways are listed in Table 3 (*p* < 0.05).

**Figure 8:**
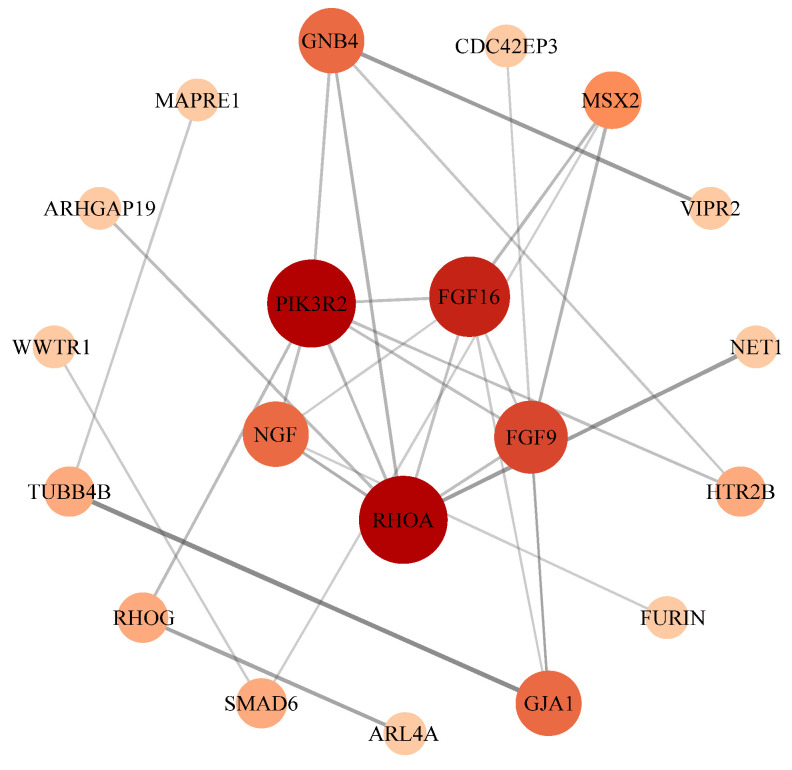
PPI Analysis of Differentially Expressed Genes in Significantly Enriched Pathways of Intramuscular Adipocytes.

To further identify key genes and pathways regulated by *FTO*, protein–protein interaction (PPI) analysis was conducted on genes from the significantly enriched pathways. As shown in Figure 8, *PIK3R2*, *FGF16*, *FGF9*, *RHOA*, and *NGF* were identified as key genes mediating *FTO*-regulated lipid accumulation in chicken intramuscular adipocytes.

### 3.3. Combined Analysis of Significantly Enriched Pathways in Subcutaneous and Intramuscular Adipocytes

**Figure 9:**
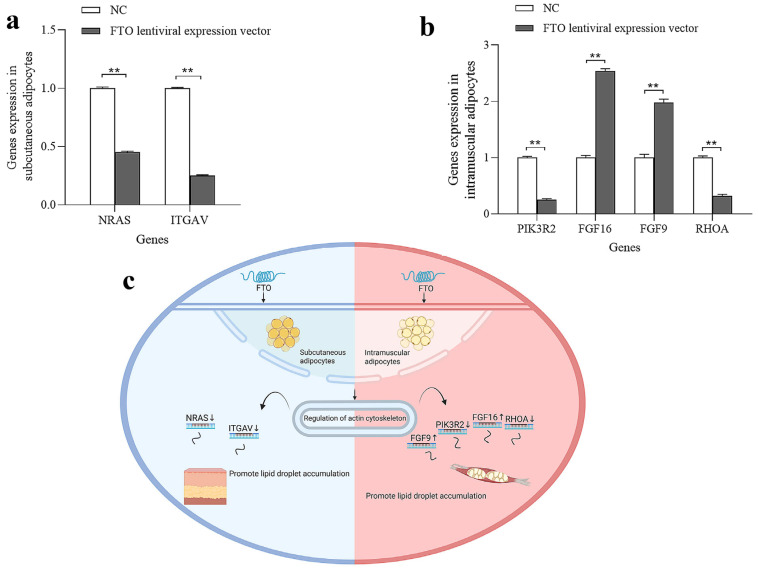
Key Genes and Pathways Responsive to *FTO* Regulation. (**a**) qPCR Validation of *FTO*-Regulated Key Genes in Subcutaneous Adipocytes; (**b**) qPCR Validation of *FTO*-Regulated Key Genes in Intramuscular Adipocytes; (**c**) Molecular pathways underlying *FTO* regulation of subcutaneous and intramuscular adipocytes; ** means *p* < 0.01.

Intersection analysis of significantly enriched pathways responsive to *FTO* regulation in subcutaneous and intramuscular adipocytes was performed. The results revealed that the regulation of the actin cytoskeleton, *MAPK* signaling pathway, and cytokine–cytokine receptor interaction were commonly enriched in both cell types. Key genes in subcutaneous adipocytes (*NRAS* and *ITGAV*) and key genes in intramuscular adipocytes (*PIK3R2*, *FGF16*, *FGF9* and *RHOA*) were significantly enriched in the regulation of the actin cytoskeleton pathway. qPCR verified the consistency of the seven DEGs identified by deep sequencing in terms of the direction of regulation and statistical significance (Figure 9a,b, *p* < 0.05). Taken together, the regulation of the actin cytoskeleton was identified as the core pathway underlying *FTO*-regulated lipid deposition in both subcutaneous and intramuscular adipocytes (Figure 9c).

## 4. Discussion

### 4.1. Regulatory Role of FTO in Lipid Deposition in Chicken Adipocytes

Based on our previous studies and published literature, we hypothesized that *FTO* may play an important role in subcutaneous and intramuscular fat deposition in chickens. Accordingly, *FTO* lentiviral expression vectors were constructed and transfected into primary subcutaneous and intramuscular adipocytes. Changes in adipocyte proliferation and lipid deposition were observed to elucidate the function of *FTO* in these two cell types. The results showed that overexpression of *FTO* significantly inhibited proliferation while markedly promoting lipid droplet accumulation in both subcutaneous and intramuscular adipocytes. In contrast, transfection of *FTO* siRNA significantly increased proliferation while markedly inhibiting lipid droplet accumulation in both cell types. These findings confirmed the role of *FTO* in regulating fat deposition. This regulatory pattern was consistent with its previously reported positive effect on abdominal fat deposition in chickens [13]. The results of the present study contribute to a deeper understanding of *FTO*’s function in regulating chicken fat-related traits and provide a foundation for further elucidating the molecular mechanisms underlying subcutaneous and intramuscular fat deposition.

### 4.2. Pathways and Genes Associated with FTO-Regulated Lipid Deposition in Chicken Subcutaneous Adipocytes

Transcriptome sequencing revealed a total of 413 differentially expressed genes (DEGs) responsive to *FTO* regulation during lipid deposition in subcutaneous adipocytes, including 98 upregulated and 315 downregulated genes. To further characterize the functions of these DEGs, KEGG enrichment analysis was performed. The results indicated that the DEGs were significantly enriched in 17 signaling pathways, including regulation of the actin cytoskeleton, endocytosis, vascular smooth muscle contraction, focal adhesion, mTOR signaling pathway, autophagy, Apelin signaling pathway, *JNK/p38 MAPK* signaling pathway, eukaryotic ribosome biogenesis, necroptosis, cytokine–cytokine receptor interaction, N-glycan biosynthesis, mitophagy, TGF-β signaling pathway, and RNA transport, all of which have been confirmed to be involved in lipid metabolism.

Among these pathways, the regulation of the actin cytoskeleton plays a critical role in lipid deposition. Park et al. reported that actin dynamics play a key role in adipogenesis by regulating morphological transformation during the differentiation of mesenchymal stem cells (MSCs) into mature adipocytes [14]. Qiu et al. demonstrated that the transferrin receptor (TFRC) regulates iron homeostasis via endocytosis, and its dysfunction leads to disturbed iron metabolism, inhibiting brown adipocyte differentiation, promoting white adipose expansion, and increasing the risk of obesity [15]. Ma et al. revealed that increased cytosolic Ca^2+^ signaling in the vascular smooth muscle contraction pathway reduces intestinal lipid levels [16]. Wei et al. found that RhoA/ROCK-mediated cytoskeletal remodeling inhibits adipogenesis and promotes osteogenic differentiation [17]. Xiong et al. reported that activation of focal adhesion enhances intramuscular adipogenesis and differentiation [18]. Li et al. demonstrated that activation of the mTOR signaling pathway increases lipid accumulation in adipose-derived stem cells [19]. Sabaté-Pérez et al. found that autophagy promotes brown adipocyte differentiation and thermogenesis, thereby inhibiting fat deposition [20]. Kim et al. reported that losartan, an angiotensin AT1 receptor antagonist, promotes white adipose browning and reduces lipid deposition by inducing Apelin-mediated AMPK activation [21]. Zhao et al. revealed that pantothenic acid (PA) alleviates lipid metabolism disorders and reduces fat deposition via the JNK/p38 MAPK pathway [22]. In ribosome biogenesis, AMPK activation reduces the expression of lipogenic proteins by inhibiting the mTOR pathway, thereby decreasing triglyceride accumulation [23]. The necroptosis regulator MLKL inhibits PPARγ activity and impedes adipogenesis in its inactive state, whereas activation of MLKL may relieve this inhibition and indirectly promote adipocyte proliferation [24]. Zhang et al. found that the cytokine IL-18 promotes thermogenesis in brown adipose tissue and enhances insulin signaling in white adipose tissue, thus improving metabolic homeostasis [25]. Liu et al. demonstrated that increased activity of core fucosyltransferase (FUT8) in the N-glycosylation pathway exacerbates adipose tissue inflammation and insulin resistance by altering the glycosylation of inflammatory factors, impairing adiponectin function, and aggravating fat deposition [26]. Cremonini et al. reported that activation of the mitophagy pathway (e.g., PINK1/Parkin) promotes white adipose browning and enhances lipolysis [27]. Ma et al. showed that TSH-stimulated hepatocyte exosomes promote triglyceride accumulation in adipocytes via the TGF-β1/ATGL axis [28]. Liu et al. found that m5C modification in RNA transport regulates preadipocyte proliferation and differentiation by controlling the nuclear export of CDKN1A mRNA [29]. Protein–protein interaction (PPI) analysis of DEGs identified CUL1, NRAS, ITGAV, GNAQ, and HSP90AA1 as key genes for *FTO*-regulated lipid deposition in chicken subcutaneous adipocytes. Among them, NRAS and ITGAV were co-enriched in the regulation of the actin cytoskeleton pathway. Ren et al. found that, as a small GTPase of the RAS family, palmitoylation of NRAS affects its membrane localization and activity, thereby regulating fatty acid uptake and lipid droplet formation [30]. Morandi et al. reported that overexpression of ITGAV in adipose-derived stem cells inhibits TAZ activity in the Hippo signaling pathway, negatively regulates the expression of adipogenic genes such as PPARγ, and thus suppresses adipocyte differentiation [31]. Collectively, *FTO* may regulate fat deposition by modulating the expression of genes (e.g., *NRAS* and *ITGAV*) within the actin cytoskeleton pathway.

### 4.3. Pathways and Genes Involved in FTO-Regulated Lipid Deposition in Intramuscular Adipocytes

A total of 164 differentially expressed genes (DEGs) responsive to *FTO* regulation were identified during lipid deposition in intramuscular adipocytes, including 71 upregulated and 93 downregulated genes. These DEGs were significantly enriched in 13 pathways, including focal adhesion, regulation of the actin cytoskeleton, MAPK signaling pathway, gap junction, cytokine–cytokine receptor interaction, FoxO signaling pathway, apoptosis, C-type lectin receptor signaling pathway, and TGF-β signaling pathway. All of these pathways have been reported to be directly or indirectly associated with fat deposition. Studies have shown that insulin stimulation induces actin cytoskeleton remodeling, promoting the translocation of the glucose transporter GLUT4 to the cell membrane, thereby enhancing glucose uptake in adipocytes. Actin depolymerizing agents disrupt this process, leading to insulin resistance. Gelsolin, an actin-severing protein, promotes insulin secretion by regulating actin filament dynamics and coordinating with the MAPK signaling pathway, indirectly affecting lipid metabolism [32]. Adipose tissue-specific knockout of the focal adhesion protein Kindlin-2 in mice results in impaired adipose development, including reduced white and brown adipose mass, accompanied by fatty liver and insulin resistance [33]. cytokine–cytokine receptor interaction is involved in the regulation of lipid metabolism. For example, IL-27 promotes white adipose browning, and its serum levels are decreased in obese individuals [34]. The FoxO signaling pathway is a central regulator of lipid metabolism. Chakrabarti et al. found that FoxO1 directly activates *ATGL* transcription to promote lipolysis, induces white adipose browning by activating *UCP1* and PGC-1α, and enhances fatty acid oxidation [35]. The C-type lectin receptor signaling pathway indirectly affects lipid metabolism. CLR-Dectin-1 is highly expressed in the subcutaneous adipose tissue of obese individuals, positively correlates with BMI, and negatively correlates with adiponectin. In high-fat diet-induced obese mice, Dectin-1 deficiency reduces inflammatory factors in adipose tissue by 40% and improves insulin sensitivity by 25% [36]. The TGF-β signaling pathway regulates lipid metabolism by modulating energy metabolism and fibrosis. The TGF-β/Smad3 axis inhibits PGC-1α expression, reduces mitochondrial biogenesis, decreases energy expenditure, and leads to fat accumulation. In high-fat diet-induced obese mice, Smad3 knockout upregulates adipose browning genes, increases mitochondrial content, improves insulin sensitivity, and reduces body weight gain [37]. Tirosh et al. found that liver-specific knockout of Cx43 in mice reduces endoplasmic reticulum stress and improves insulin sensitivity, glucose tolerance, and lipid metabolism, suggesting that gap junctions indirectly regulate lipid metabolism by affecting hepatic ER stress [38].

To further identify key genes and pathways responsive to *FTO* regulation, protein–protein interaction (PPI) analysis was performed on differentially expressed genes (DEGs) in significantly enriched pathways. The results showed that *PIK3R2*, *FGF16*, *FGF9*, *RHOA*, and *NGF* were identified as key genes for *FTO*-regulated lipid deposition in chicken intramuscular adipocytes. Among them, *PIK3R2*, *FGF16*, *FGF9* and *RHOA* were enriched in the regulation of the actin cytoskeleton pathway. Studies have demonstrated that *PIK3R2*, *FGF16*, *FGF9*, and *RHOA* are directly or indirectly involved in fat deposition. *PIK3R2* promotes the expression of cell cycle-related proteins, such as Cyclin D1, by activating the PI3K-AKT pathway, thereby regulating cell proliferation. During adipocyte differentiation, PIK3R2 enhances the activity of PPARγ and C/EBPα by phosphorylating transcription factors, including FOXO1, thus promoting the differentiation of preadipocytes into mature adipocytes [39]. Mice deficient in PIK3R2 exhibit elevated AMPK phosphorylation in muscle and adipose tissue, promoting fatty acid oxidation and reducing fat accumulation [40]. As a member of the fibroblast growth factor (FGF) family, FGF16 regulates cell proliferation, differentiation, and energy metabolism by activating receptors such as FGFR4. Overexpression of FGF16 significantly promotes lipid droplet accumulation and triglyceride synthesis in goat intramuscular adipocytes, whereas knockdown reduces lipid deposition and downregulates adipogenic genes [41]. In high-fat diet-induced obese mice, local injection of FGF9 increases the proportion of UCP1-positive beige adipocytes in inguinal white adipose tissue from 5% to 18% and reduces lipid deposition by 25% [42]. Inhibition of *RHOA* alleviates adipose inflammation and obesity in high-fat diet-induced obese mice [43]. The RhoA/ROCK pathway suppresses adipocyte differentiation by regulating PPARγ and actin remodeling [44]. In summary, *PIK3R2*, *FGF16*, *FGF9*, and *RHOA* play important roles in lipid metabolism in both humans and animals, serving as key genes responsive to *FTO*-regulated intramuscular fat deposition in chickens.

To further identify key genes and pathways responsive to *FTO*-regulated lipid deposition, intersection analysis was performed on the significantly enriched pathways in subcutaneous and intramuscular adipocytes. The results revealed that the *MAPK* signaling pathway, cytokine–cytokine receptor interaction, regulation of the actin cytoskeleton, and Salmonella infection were co-enriched pathways. Among these, regulation of the actin cytoskeleton was identified as the core pathway shared by both subcutaneous and intramuscular adipocytes during lipid deposition. In subcutaneous adipocytes, *NRAS* and *ITGAV* were key genes, both of which were enriched in this pathway. In intramuscular adipocytes, genes enriched in this pathway included *ITGA1*, *FGF9*, *RHOA*, *PDGFB*, *PIK3R2*, and *FGF16*, with *FGF9*, *PIK3R2*, *FGF16*, and *RHOA* acting as key genes. These findings suggest that the *FTO* regulates lipid deposition in chicken subcutaneous and intramuscular adipocytes by modulating the expression of different genes within the regulation of the actin cytoskeleton pathway.

## 5. Conclusions

*FTO* inhibits the proliferation of both subcutaneous and intramuscular adipocytes while promoting fat deposition. *FTO* differentially regulates lipid deposition in chicken subcutaneous and intramuscular adipocytes by targeting genes involved in the regulation of the actin cytoskeleton pathway. Specifically, *NRAS* and *ITGAV* serve as key genes for subcutaneous fat deposition, while *FGF9*, *PIK3R2*, *FGF16*, and *RHOA* act as key genes for intramuscular fat deposition.

## Figures and Tables

**Table 1:** Primers of Genes Used in the Real-Time PCR.

GENES	SEQUENCE 5′ TO3 ′
*β-ACTIN*	F: 5′GTCCACCTTCCAGCAGATGT3′
	R: 5′ATAAAGCCATGCCAATCTCG3′
*FTO*	F: 5′CTGGTCCTCCAGAAGTTCG3′
	R: 5′CTGCTCTTCTGGCAAGCTCT3′
*NRAS*	F: 5′CCAGCTCATCCAGAACCACTT3′
	R: 5′TCCTGCAGTGTCCAGAATGTC3′
*ITGAV*	F: 5′GACGCCTCCTCGATGTTTCT3′
	R: 5′ATACAATGGGGCACAAGCCA3′
*FGF9*	F: 5′TTTATTGGCCACGTGAGGCT3′
	R: 5′GCCACAAAGTAAAGCCCAGC3′
*FGF16*	F: 5′CACTTTTTACCGAGGCCCGT3′
	R: 5′TGGGAAAAAGCTTGGGAGCC3′
*RHOA*	F: 5′CCTGTGGAGCAGGAAGCG3′
	R: 5′AAGCCAACTCCACCTGCTTT3′
*PIK3R2*	F: 5′TTCGGGGAGGTTCGTTCTTC3′
	R: 5′TCCAGCCCGTTCCTTCTTTC3′
FTO-205	S: 5′CCAGAUAUUCCAAGCUAAUTT3′
	A: 5′AUUAGCUUGGAAUAUCUGGTT3′
FTO-858	S: 5′GCUGAAGAAGCUACUGAUUTT3′
	A: 5′AAUCAGUAGCUUCUUCAGCTT3′
FTO-1286	S: 5′GCUUAAGCCUAUGGCUAAATT3′
	A: 5′UUUAGCCAUAGGCUUAAGCTT3′

**Table 2:** Significantly Enriched Pathways of Differentially Expressed Genes in Subcutaneous Adipocytes.

Pathway	*p* Value	Genes
Regulation of actin cytoskeleton	0.0006	*LPAR4*, *ITGAV*, *NRAS*, *ITGA4*, *ITGB8*, *PDGFRA*, *BRAF*, *ROCK1* *CRKL*, *PPP1R12A*, *ROCK2*
Endocytosis	0.0008	*RAB22A*, *RAB10*, *PARD6B*, *DAB2*, *CHMP1A*, *CHMP6*, *STAM*, *EEA1* *PDGFRA*, *ARAP2*, *IGF2R*, *ARHGEF3*
Vascular smooth muscle contraction	0.0013	*EDN3*, *EDNRA*, *ROCK1*, *BRAF*, *MRVI1*, *PPP1R12A*, *ROCK2*, *GNAQ*
Focal adhesion	0.0021	*ARHGAP5*, *ITGAV*, *ITGA4*, *ITGB8*, *PDGFRA*, *BRAF*, *ROCK1*, *CRKL*, *PPP1R12A*, *ROCK2*
mTOR signaling pathway	0.0036	*RPS6KA3*, *RICTOR*, *CAB39*, *FZD1*, *ATP6V1D*, *BRAF*, *NRAS*, *FNIP2*
Autophagy—animal	0.0050	*UVRAG*, *RB1CC1*, *NRAS*, *EIF2AK4*, *RAB33B*, *GABARAPL1*, *IRS4*
Apelin signaling pathway	0.0055	*NOV*, *GNB4*, *NRAS*, *GNB*, *GABARAPL1*, *PIK3CG*, *GNAQ*
MMAPK signaling pathway	0.0066	*RPS6KA3*, *RASA2*, *NGF*, *ATF2*, *PDGFRA*, *BRAF*, *NRAS*, *CRKL*, *IL1R1*, *STK4*, *MAP4K3*
Ribosome biogenesis in eukaryotes	0.0073	*SBDS*, *BMS1*, *HEATR1*, *XRN1*, *XPO1*
Necroptosis	0.0089	*SMPD1*, *CHMP1A*, *CHMP6*, *HSP90AA1*, *PARP4*, *JAK2*, *FADD*
cytokine–cytokine receptor interaction	0.0096	*TNFSF15*, *IL18*, *NGF*, *CXCR7*, *BMPR2*, *EDA*, *INHBA*, *IL1R1*, *CCL4*
N-Glycan biosynthesis	0.0120	*ALG2*, *MAN2A1*, *B4GALT2*, *B4GALT1*
Mitophagy-animal	0.0204	*GABARAPL1*, *NRAS*, *TAX1BP1*, *USP15*
TGF-beta signaling pathway	0.0221	*INHBA*, *CUL1*, *ROCK1*, *BMPR2*, *DCN*
RNA transport	0.0293	*THOC2*, *TACC3*, *UBE2I*, *TPR*, *EIF1B*, *XPO1*
Salmonella infection	0.0360	*CCL4*, *IL18*, *ROCK1*, *ROCK2*
Hedgehog signaling pathway	0.0375	*EVC*, *CUL1*, *SPOPL*

**Table 3:** Significantly Enriched Pathways of Differentially Expressed Genes in Intramuscular Adipocytes.

Pathway	*p* Value	Genes
MAPK signaling pathway	0.014	*HGF*, *FGF9*, *PDGFB*, *NGF*, *AKT3*, *FGF16*
cytokine–cytokine receptor interaction	0.018	*IL18*, *NGF*, *BMP15*, *IFNGR2*, *IL4R*
FoxO signaling pathway	0.014	*CDKN2B*, *CDKN2A*, *PIK3R2*, *AKT3*, *FOXO4*
TGF-beta signaling pathway	0.031	*CDKN2B*, *CDKN2A*, *SMAD6*, *RHOA*
Focal adhesion pathway	0.003	*HGF*, *ITGA1*, *RHOA*, *PDGFB*, *PIK3R2*, *AKT3*
Regulation of actin cytoskeleton	0.003	*ITGA1*, *FGF9*, *RHOA*, *PDGFB*, *PIK3R2*, *FGF16*
Gap junction	0.001	*TUBA4A*, *HTR2B*, *TUBB4B*, *PDGFB*, *GJA1*
Apoptosis	0.016	*TUBA4A*, *NGF*, *PIK3R2*, *AKT3*
Toll-like receptor signaling pathway	0.026	*TLR5*, *PIK3R2*, *AKT3*
C-type lectin receptor signaling pathway	0.029	*RHOA*, *PIK3R2*, *AKT3*
Salmonella infection	0.002	*TLR5*, *IFNGR2*, *IL18*, *RHOG*
Influenza A	0.019	*IFNGR2*, *IL18*, *PIK3R2*, *AKT3*
AGE-RAGE signaling pathway in diabetic complications	0.031	*PIM1*, *PIK3R2*, *AKT3*

## Data Availability

The original contributions presented in this study are included in the article. Further inquiries can be directed to the corresponding author.
